# Supplementary figures and images for: Metabolomic and Transcriptomic Analyses Revealed Lipid Differentiation Mechanisms in Agaricus bisporus at Ambient Conditions
Source: J Fungi (Basel). 2024 Jul 30;10(8):533. doi: 10.3390/jof10080533 (PMC11355595; doi:10.3390/jof10080533)

A

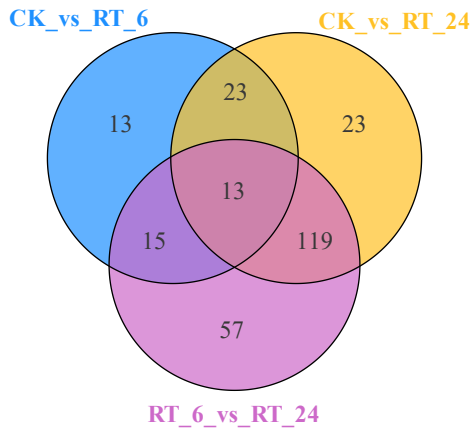

B

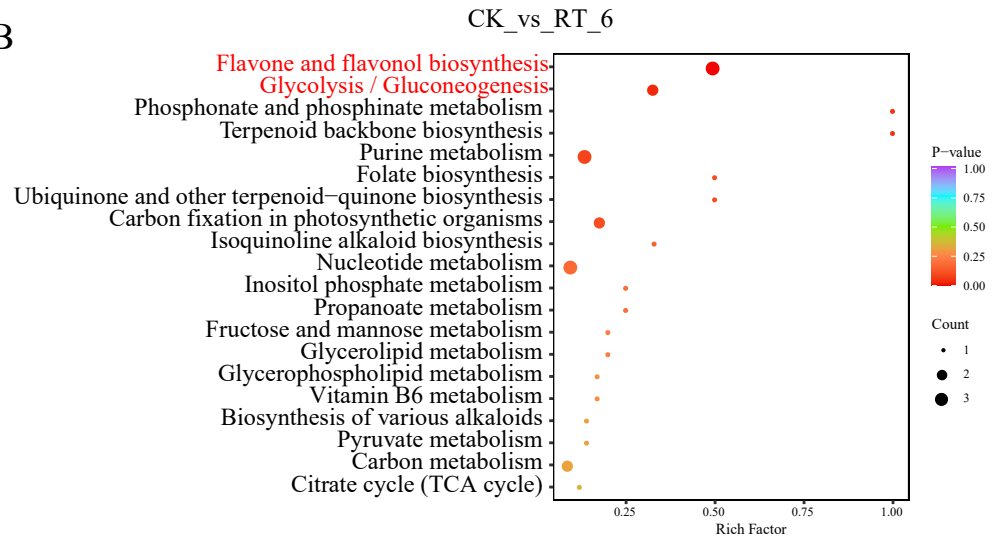

C

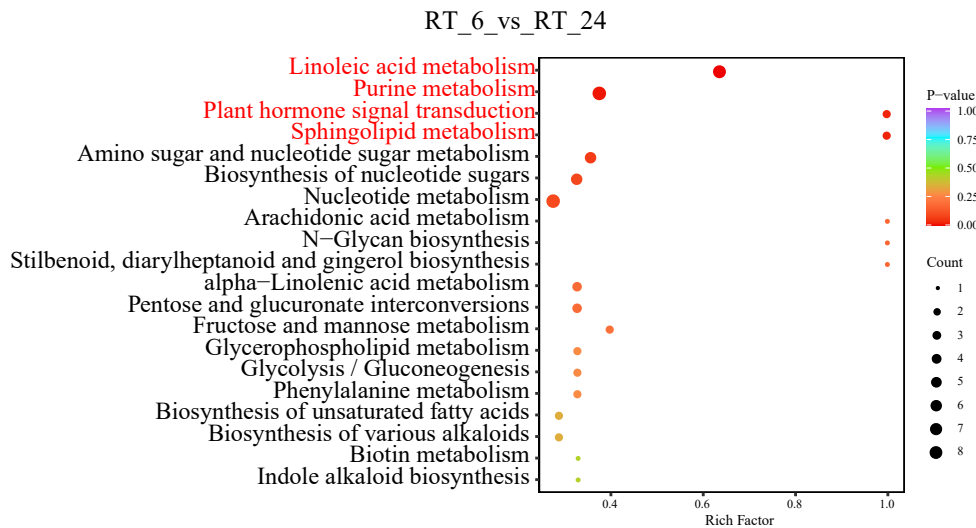

D

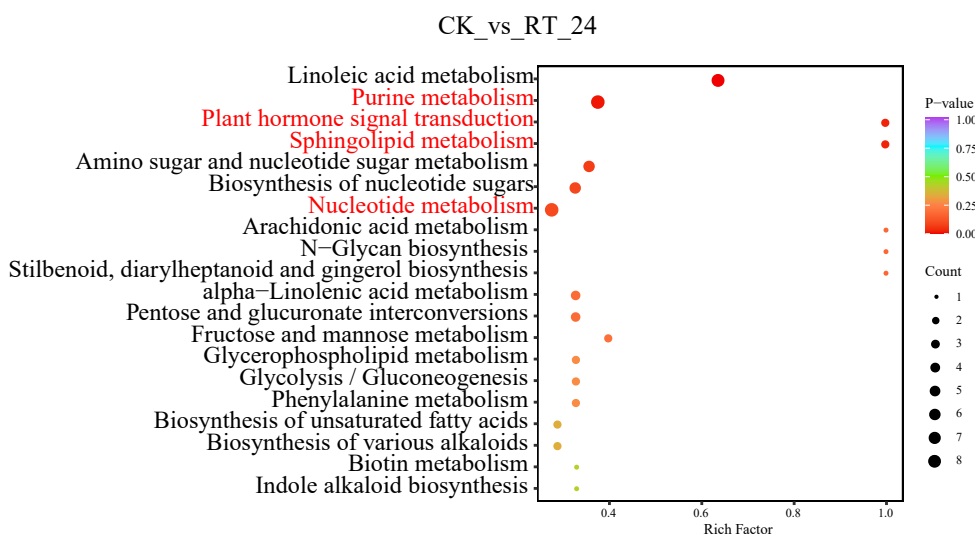

Supplement: Supplementary file 1 [file jof-10-00533-s001.zip › Supplementary Material/Figure S1.pdf]

A

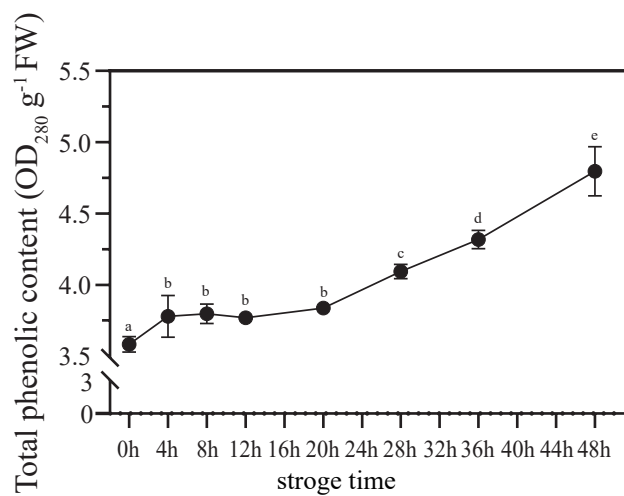

B

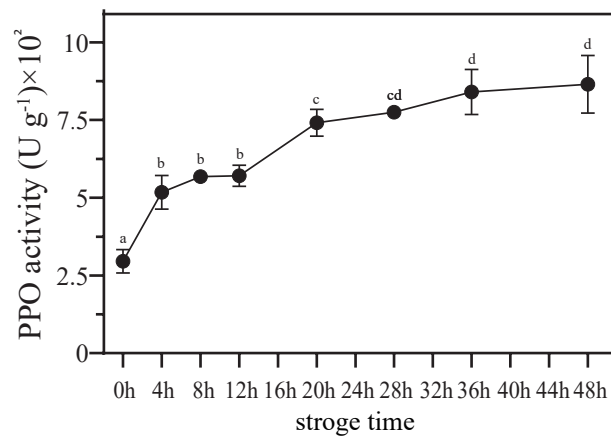

C

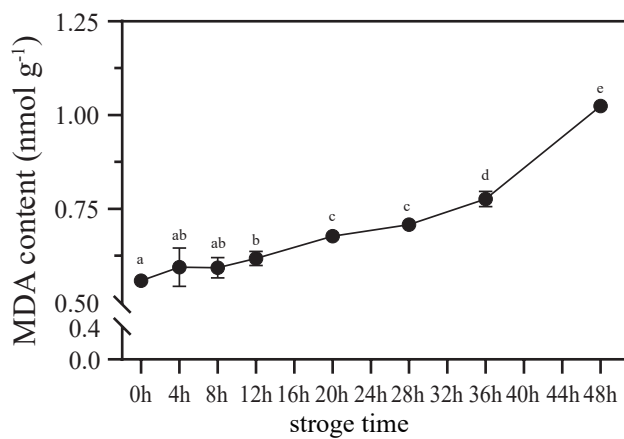

D

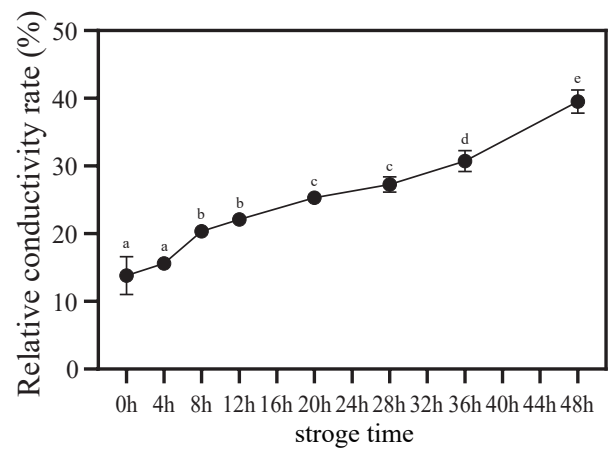

Supplement: Supplementary file 1 [file jof-10-00533-s001.zip › Supplementary Material/Figure S2.pdf]

A

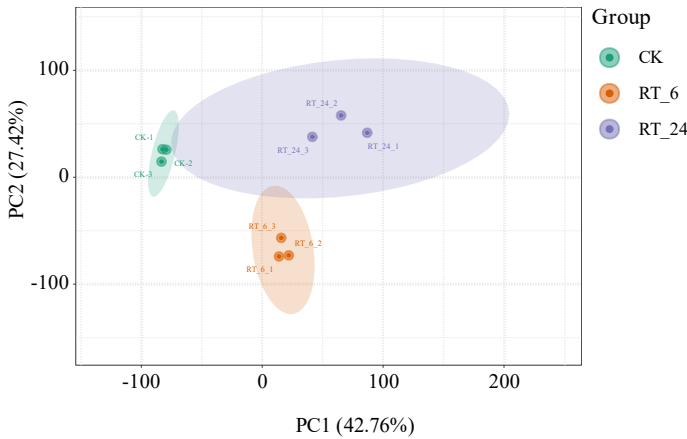

B

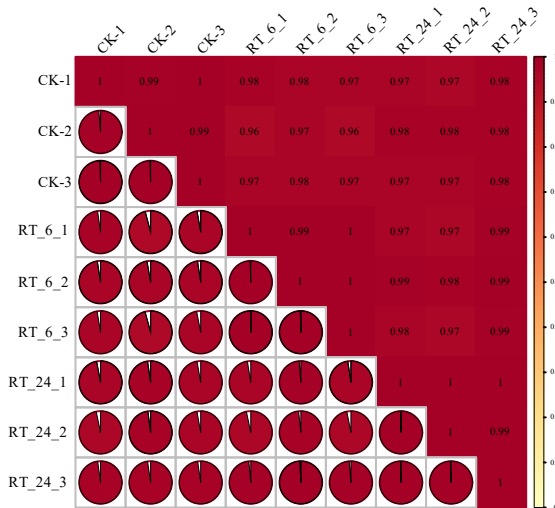

Supplement: Supplementary file 1 [file jof-10-00533-s001.zip › Supplementary Material/Figure S3.pdf]

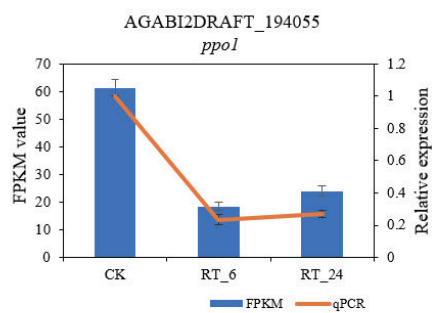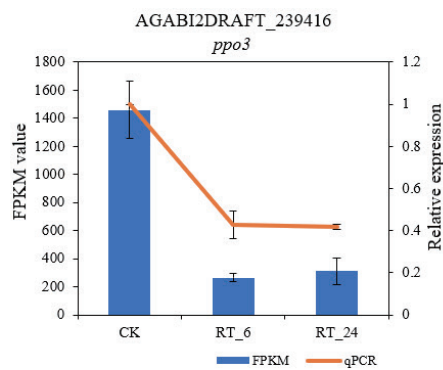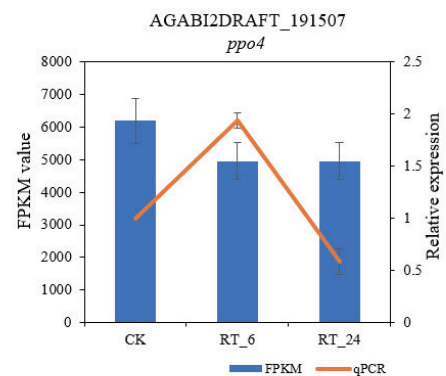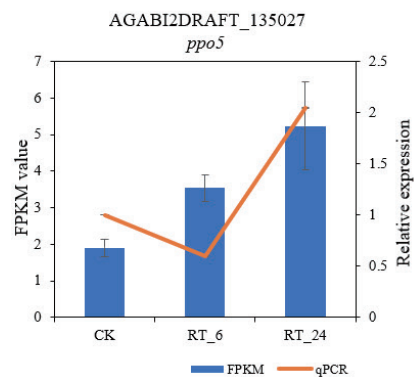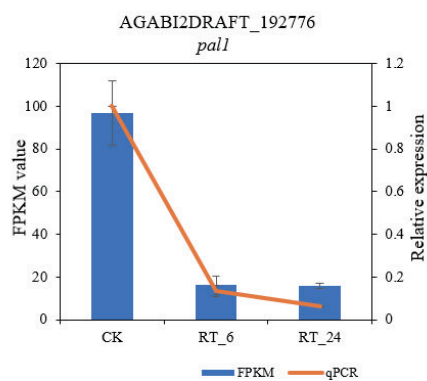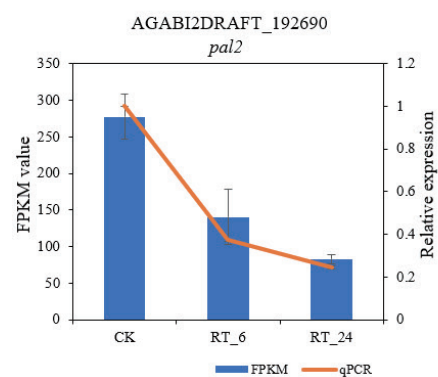

Supplement: Supplementary file 1 [file jof-10-00533-s001.zip › Supplementary Material/Figure S4.pdf]
